# Chemical Composition and Insecticidal Potential of Six Essential Oils from Morocco against *Dactylopius opuntiae* (Cockerell) under Field and Laboratory Conditions

**DOI:** 10.3390/insects12111007

**Published:** 2021-11-09

**Authors:** Chaimae Ramdani, Karim El Fakhouri, Mohamed Sbaghi, Rachid Bouharroud, Rachid Boulamtat, Abderrahim Aasfar, Abdelhalim Mesfioui, Mustapha El Bouhssini

**Affiliations:** 1 Entomology Laboratory, International Center for Agricultural Research in the Dry Areas (ICARDA), Rabat Institutes, Rabat 10100, Morocco; k.el-fakhouri@cgiar.org (K.E.F.); r.boulamtat@gmail.com (R.B.); 2 Laboratory of Biology and Health, Department of Biology, Faculty of Science, Ibn-Tofail University, Kenitra 14000, Morocco; a.mesfioui@yahoo.fr; 3 National Institute of Agronomic Research (INRA), Rabat 10100, Morocco; msbaghi@yahoo.fr; 4 Integrated Crop Production Unit, Regional Center for Agronomic Research of Agadir (INRA), Avenue des FAR, Inezgane 80000, Morocco; rachid.bouharroud@inra.ma; 5 Green Biotechnology Laboratory, Moroccan Foundation for Advanced Science, Innovation and Research (MAScIR), Rabat Design Centre, Rue Mohamed Al Jazouli, Madinate Al Irfane, Rabat 10100, Morocco; a.aasfar@mascir.ma; 6 AgroBioSciences Research Division, Mohammed VI Polytechnic University, Lot 660, Hay Moulay Rachid, Benguerir 43150, Morocco; Mustapha.ElBouhssini@um6p.ma

**Keywords:** wild cochineal, essential oils, black soap, toxicity, chemical compound, cactus pear

## Abstract

**Simple Summary:**

The prickly pear *Opuntia ficus-indica* L. has an important economic role as a source of food for human consumption, for cosmetic and pharmaceutical purposes, and as fodder for livestock in Morocco. This crop is now attacked by a new sap-sucking insect pest, the wild-scale *Dactylopius opuntiae* (Cockerell). The aim of this study was to investigate the insecticidal potential of essential oils as alternative to synthetic insecticides for the control of *D. opuntiae* nymphs and adult females under laboratory and field conditions. Results revealed that *Mentha pulegium* and *Origanum vulgare* oils provided a rich source of bioactive compounds and exhibited high contact toxicity against *D. opuntiae*. In addition, *M. pulegium* and *O. vulgaris* oils applied in combination with black soap induced the highest toxic activity on adult females under field conditions.

**Abstract:**

The carmine cochineal *Dactylopius opuntiae* (Cockerell) is the major insect pest of the prickly-pear cactus *Opuntia ficus-indica* (L.) in Morocco. The present study investigated the insecticidal activities of six essential oils (EOs) against nymphs and adult females of *D. opuntiae* applied singly or in combination with a detergent under laboratory and field conditions. Under laboratory conditions, *M. pulegium* and *O. vulgare* L. essential oils showed a high level of insecticidal activity at 5%, with 98% and 92% females’ mortality, respectively, 5 days after treatments. The *M. pulegium* and *O. vulgaris* oils at 5% applied in combination with black soap at (60 g/L) induced the highest toxic activity on adult females, 100% and 96% at 5 days after treatments, respectively. Under field conditions, *M. pulegium* and *O. vulgare* oils at 5% in combination with black soap (60 g/L) showed the highest adult female mortalities with 96.33 and 92.56%, respectively, 7 days after the first application. The double application of *M. pulegium* oil at 5% significantly increased the mortality of adult females up to 91%, 5 days after the second spray. GC-MS analysis revealed that the most abundant constituent of *M. pulegium* and *O. vulgare* oils was pulegone (84.69%) and durenol (76.53%), respectively. These findings showed that the use of *M. pulegium* and *O. vulgare* in combination with black soap or in double sprays could be incorporated in the management package for the control of the wild cochineal *D. opuntiae*, as a safe and natural alternative to chemical insecticides.

## 1. Introduction

The prickly pear, *Opuntia ficus-indica* (L.) Mill. (Caryophyllales: Cactaceae), is a plant originating from Mexico and belonging to the Cactaceae family. In Morocco, the cactus was introduced in 1770 and it is now well represented throughout the national landscape [1]. The cactus area increased from 45,000 ha in the early 1990s to over 150,000 ha in 2017 [2].

Cactus pear plays an important economic role as a source of animal feed and food for human consumption, in addition to the production of various cosmetic and pharmacological preparations. The cactus crops are gaining increasing interest as an ecological crop in preventing soil erosion and preserving biodiversity. It has been threatened recently by the wild cochineal *Dactylopius opuntiae* (Hemiptera: Dactylopiidae), which was introduced in Morocco in 2014 [3]. The cochineal has spread quickly and invasively in the other regions of Morocco, causing massive economic losses. Nymphs and adult females of *D. opuntiae* feed directly on plants by sucking sap on the cladode, causing drying, weakening, and eventually death of the plant host [4]. Several promising integrated pest management options (host plant resistance, biological control, and biopesticides) for the control of the cochineal are being developed in Morocco [5,6,7,8,9].

To alleviate problems associated with the use of chemical pesticides to control the wild cochineal, a sustainable alternative is necessary. The pool of plants possessing insecticidal substances such as botanical extracts and essential oils is enormous and may provide an alternative approach in controlling insect pests [10,11,12,13,14]. These have generated strong interest in recent years as potential sources of natural insecticides with lower risks for human health and the environment, and less impact on natural enemies [15,16]. Several authors have reported high insecticidal effects of botanical extracts, vegetable oils, essential oils, detergent, soaps, and other bioactive substances to control the carmine cochineal in prickly pear [17,18,19,20,21]. 

Essentials oils are natural volatiles and fragrant compounds produced as secondary metabolites. They have been recognized to possess various biological proprieties including fungicidal, bactericidal, acaricidal, or insecticidal [22,23,24,25]. Several chemical compounds of the plant essential oils, consisting of flavonoids, terpenoids, alkaloids, steroids and phenols, sesquiterpene, and other compounds, have been reported as having insecticidal potential against different insect pests [26,27]. In this context, several researchers have reported the efficiency of essential oils and terpenoids for the control of *D. opuntiae*. Vázquez-García et al. [20] found that different essential oils belonging to various families, including *Ocimum basilicum* L., *Mentha spicata* L., *Cymbopogon winterianus* and *Lippia graveolens* Kunth, showed an important insecticide activity against 1st instar *D. opuntiae*. Similarly, Pérez-Ramirez et al. [28] reported that the terpenoids such as eugenol, 1,8-cineol, and menthol had significantly reduced the number of *D. opuntiae* nymphs. Besides, these secondary metabolites have shown a range of biological activities, such as contact toxicity [29,30] and fumigant toxicity [31,32]. They can also cause repellency [33,34], antifeedant effects [35], growth effect, and inhibition of reproduction [36,37]. 

The present work aims to study the insecticidal proprieties of several essential oils for the control of different stages of *D. opuntiae* under laboratory and field conditions, and to identify the chemical composition of the most bioactive EOs using gas chromatography–mass spectrometry (GC-MS) analysis. The results of this study will be exploited for the development of new formulations based on bioactive compounds potentially useful for the protection of the cactus plants against the wild cochineal *D. opuntiae*.

## 2. Materials and Methods

### 2.1. Essential Oils

Six species of medicinal and aromatic plant species of *Mentha pulegium*, *Origanum vulgare*, *Rosmarinus officinalis*, *Thymus vulgaris*, *Myrtus communis,* and *Eucalyptus globulus* were collected from the wild area of the middle Atlas Mountain in Morocco. In the ICARDA Entomology Laboratory at Rabat, leaves of plants were rinsed in water and air dried in the shade at room temperature (26 ± 2 °C) for 5 days and stored in darkness until distillation. EOs were obtained from plants by hydrodistillation using the Clevenger apparatus [38]. For each plant, 100 g of leaves were distilled in 1000 mL of water (1:10 plant material/water volume ratio) in a flask of 1500 mL for 3 h. The extracts were dried over anhydrous sodium sulfate and stored in a brown bottle at 4 °C until further analysis and use.

### 2.2. Laboratory Bioassays

#### 2.2.1. Insect Rearing

Healthy young cladodes of prickly pear cactus (*Opuntia ficus indica*) were planted in plastic pots (27 cm in diameter by 24 cm in height) with a mixture of one-third soil, one-third sand, and one-third peat. These plants were grown in the greenhouse at 30 °C ± 2 and then exposed to heavily infested cladodes collected from the Marchouch region (33°56′10″ N 6°69′21″ W). Each infested cladode was placed between two pots, and after 20 days of the exposure, the successful artificial infestation occurred with colonies showing mature females that are carefully selected for using in various bioassays.

#### 2.2.2. Contact Toxicity

The contact toxicity effect of six essential oils was evaluated by foliar spray, at constant laboratory conditions of 26 ± 2 °C, 70 ± 5% relative humidity, and a photoperiod of 14:10 (L:D). Four concentrations (0.625, 1.25, 2.5, and 5%) were used, mixing oils with water containing 0.1% TritonX-100. Control nymphs and females of the cochineal were treated with water containing 0.1% Triton X-100. The bioassays were conducted in a completely randomized design (CRD) with five replicates per concentration for each treatment.

Ten females of the same age and 10 first instar nymphs of *D. opuntiae* were deposited separately on pieces of cladodes of the same size, using an entomological brush placed in Petri dishes (9 cm in diameter). Mortality of adult females was calculated every 24 h for 8 days after applying different treatments using the binocular microscope (MoticDM-143). Nymphs mortality was recorded after 3, 6, 24, and 72 h. The dead females revealed dark brown color and desiccation of their bodies, while the dead nymphs came off easily and had color modifications.

#### 2.2.3. Toxicity of Essential Oils in Combination with Black Soap on *D. opuntiae* Nymphs and Adult Females

The insecticidal activity of the most effective EOs was tested in combination with black soap by foliar spray using a completely randomized design with five replicates in the laboratory. The highest concentration of the tested oils (5%) was used for its efficacy against *D. opuntiae* nymphs and mature females. The Moroccan detergent black soap at 60 g/L, manufactured from natural fatty acids derived from olive oil, was selected for its role in degradation of the cuticular wax [9]. The soap solution was sprayed first on cladodes of the same size followed by EOs using a 1 L hand sprayer. Mortality of nymphs was recorded 3, 6, and 24 h after treatments, and adult females’ mortality was registered every 24 h for 8 days after treatments.

### 2.3. Field Bioassay

The promising EOs that showed significant toxicity against *D. opuntiae* females and nymphs under laboratory conditions were selected to assess their efficacy in natural field infestations during October–November 2020. The field trial was conducted close to ICARDA’s Marchouch Research Station, Rabat, Morocco (34°52′33″ N 6°32′07″ W).

The trial was conducted in a randomized complete block with three replicates, and four cladodes were selected on each plot. Treatments included most effective essential oils selected from laboratory trials, *M. pulegium, O. vulgare*, *R. officinalis*, and *M. communis*. The four EOs were applied on cladodes alone at concentration of 5%, and on cladodes previously sprayed with black soap at (60 g/L). The cladodes treated with water mixed to Triton X-100 served as control. EOs solutions were mixed with the Triton X-100-stabilized emulsion used to facilitate the mixing of oils with water before treatment applied using a 2 L hand sprayer. Mortality of nymphs and adult females was recorded 24 h before spray and 1, 3, 5, and 7 days after the first and second sprays on three infested cladodes selected for each treatment. The treatments were applied on selected cladodes at the medium levels of infestation (26–50%) using a rating scale modified from [39].

### 2.4. GC-MS Analysis Conditions

Analysis of the four selected bioactive EOs (*O. vulgare*, *M. pulegium*, *R. officinalis*, and *M. communis*) was performed using gas chromatography (GC) (Agilent 7890A Series) coupled to mass spectrometry (MS) equipped with a multimode injector and a 123-BD11 column of dimension (15 m × 320 μm × 0.1 μm) at Moroccan Foundation for Advanced Science, Innovation and Research (MAScIR) Institute. Ten µL of the liquid samples were dissolved in an appropriate volume of chloroform. Four µL of the soluble extract was injected into the column by 1:5 split mode using helium as carrier gas at a flow rate of 2 mL min^−1^. The composition of the essential oil determined from the peak areas was calculated as a percentage of the total compounds existing in the sample detection using full scan mode between 30–1000 *m/z*, with gain factor of 5 and electron impact ionization. The temperatures of the ion source and the quadrupoles were 230 and 150 °C, respectively. The oven temperature was programmed at 30 °C for 3 min and then increased by 10 °C min^−1^ to 250 °C. The compounds identification was carried out using the NIST 2017 MS Library.

### 2.5. Data Analyses 

Mortality percentages were transformed into angular values (arcsine √P) before the statistical analysis. The transformed percentages were subjected to two-way analysis of variance (ANOVA) (oil concentration and oil source). The means were separated by Tukey’s test (*p* < 0.05) using Genstat (20th Edition, VSN International, Hemel Hempstead, UK).

## 3. Results

### 3.1. Laboratory Bioassays

#### 3.1.1. Effect of the Essential Oils on Adult Females and Nymphs of *D. opuntiae*

The mortality of nymphs and adult females of *D. opuntiae* after exposure to EOs is presented in Table 1 and Table 2. Data analysis showed a significant difference (*p* < 0.001) in mortality of nymphs and adult females *D. opuntiae*, caused by the six EOs at different tested concentrations for various exposure times. At 3 days after application, the highest percentage mortality (74%) of adult females was recorded for *M. pulegium* oil at 5%, followed by *O. vulgare* oil (66%) at 5%. Among all EOs tested, the highest mortality rates of *D. opuntiae* females were recorded by *M. pulegium* and *O. vulgare* with 98 and 92% at 5%, respectively (5 days after treatment). The increasing concentrations of the EOs significantly increased the mortality of females that were exposed to different essential oils for different periods. However, the highest concentration of *R. officinalis* L, *T. vulgaris* L., and *M. communis* L. oils had caused important mortality rates (82%) and (80%) and (80%) at 8 days after application, respectively, while no mortality was observed by *E. globulus* oil against *D. opuntiae* females at all concentrations tested.

Data analysis showed a significant difference in nymph mortality percentage among all the tested oils during the different exposure periods (*p* < 0.001). At 3 h after application, the highest percentage mortality (86%) of nymphs was recorded for *O. vulgare* oil at 5%, followed by *T. vulgaris* and *M. pulegium* oils with (78%) and (74%) at 5%, respectively. At 24 h after application, *M. pulegium* and *O. vulgare* oils showed the highest levels of nymph mortality (98%) at 5%, respectively. At 72 h after application, the highest percentage mortality (100%) of nymphs was recorded for the *T. vulgaris* oil at 5%. The *O. vulgare* and *M. pulegium* oils at 5% were the second most effective oils, both resulting in 98% nymph mortality.

#### 3.1.2. Insecticidal Effects of EOs in Combination with Black Soap on *D. opuntiae* Nymphs and Adult Females

**Figure 1:**
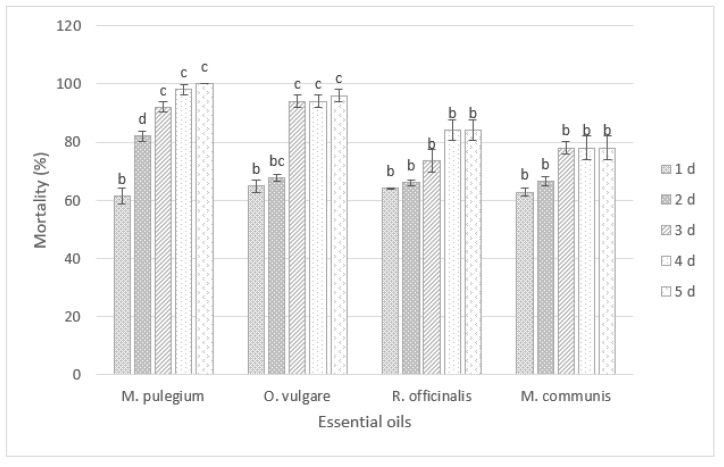
Insecticidal effects of different essential oils in combination with black soap on *D. opuntiae* adult females after 5 days of application. The different letters indicate significant differences between groups based on Tukey test (*p* < 0.05).

**Figure 2:**
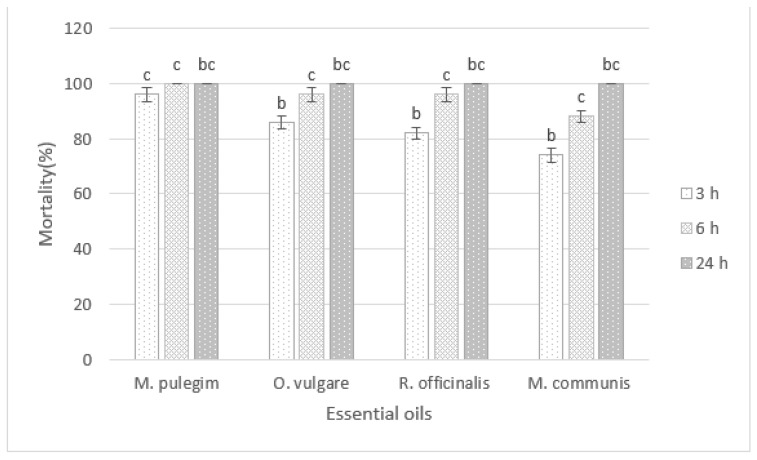
Insecticidal effects of different essential oils in combination with black soap on *D. opuntiae* nymphs after 24 h of application. The different letters indicate significant differences between groups based on Tukey test (*p* < 0.05).

The mortality of *D. opuntiae* nymphs and adult females after exposure to four EOs treatments (*M. pulegium*, *O. vulgare*, *M. communis*, *R. officinalis)* at 5% in combination with black soap (60 g/L) for various exposure times is presented in Figure 1 and Figure 2. 

The ANOVA showed significant differences in mortality of adult females caused by different treatments through various exposure periods (Figure 1). At 5 d after treatments, the *M. pulegium* and *O. vulgaris* oils at 5% combined with black soap (60 g/L) induced the highest toxic activity on *D. opuntiae* adult females among all tested extracts, with a percentage mortality of 100% and 96%, respectively.

Data analysis showed a significant difference in nymph mortality percentage among all the tested oils during the different exposure periods (*p* < 0.001). At 3 h post-application, the application of the black soap (60 g/L) followed by *M. pulegium* oil at 5% showed the highest mortality (96%) of nymphs among all tested treatments. After 24 h, the highest mortality (100%) of nymphs occurred for all tested treatments.

#### 3.1.3. Chemical Components of the Four Essential Oils

Table 3 shows the chemical composition of the most effective four essential oils from leaves expressed in percentage of the total area of peaks separated by GC and identified by mass spectroscopy. The total compounds identified of *M. pulegium*, *O. vulgare*, *R. officinalis,* and *M. communis* were 92.22%, 96.46%, 97.32%, and 98.27%, respectively. The major component of *M. pulegium* was pulegone (84.69%); multiple other constituents were detected, but at low percentage, i.e., piperitenone (2.05%), humulene (2.02%), and limonene (1.69%). For *O. vulgare*, the major constituents were durenol (76.53%) and gamma-terpinene (10.25%), while eucalyptol (45.16%) was the main compound of *R. officinalis* oil, followed by 2-bornanone, with 24.74%. eucalyptol was also the dominant compound of *M. communis* oil, with 42.6%. Other compounds identified from *M. communis* oil were myrtenyl acetate and (-)-β-pinene, with 18.23% and 16.94%, respectively.

### 3.2. Field Bioassay

The mortality of *D. opuntiae* nymphs treated by EOs and their combination with black soap is presented in Table 4. Data analysis indicated a significant difference in mortality of nymphs caused by EOs applied alone at 5% and their combination with black soap at 60 g/L after the first and the second treatments (*p* < 0.001).

At 1 day after application, *M. pulegium* oil applied singly at 5% or combined with black soap (60 g/L) showed the highest rates of nymph mortalities with 90 and 87.78%, respectively (Table 4). At 5 days, the first application of *M. pulegium* alone and in combination with black soap recorded the highest nymph mortalities, with 98.89 and 100%, respectively. At 1 day after the second application, the nymph mortalities reached 100% for *M. pulegium* and *O. vulgaris* oils and their combination with black soap. At 3 days after the second spray, all treatments were statistically equal, except for *M. communis* oil sprayed alone, which reached (93.33%) of nymph mortality.

The ANOVA showed a significant difference in mortality of *D. opuntiae* adult females caused by different EOs and their combination with black soap for various exposure times for first and second treatment (*p* < 0.001; Table 5). At 1 day after first treatments, *M. pulegium* at 5% applied in combination with black soap showed the highest mortality of females (76.33%) followed by *O. vulgare* (73.30%) (Table 5). However, the application of *M. pulegium* and *O. vulgare* oils without detergent caused lower mortality rates, with 25.11 and 22.89%, respectively. The adult females’ mortalities increased significantly at 7 days after first application for *M. pulegium* (96.33%) and *O. vulgare* (92.56%) at 5% applied in combination with black soap.

Female mortality increased to 100% 3 days after the second treatment of *M. pulegium* and *O. vulgare* at 5% applied with the black soap (60 g/L), followed by *M. pulegium* at 5% without detergent and *R. officinalis* combined with the black soap, with 84.78 and 84.56%, respectively. The adult females’ mortality increased significantly 7 days after the second application for *M. pulegium* at 5%, to reach 91%. However, the moderate percentage mortalities (64.78%) and (62.56%) occurred at 7 days after the second spray for *R. officinalis* and *M. communis* at 5% applied without black soap.

## 4. Discussion

The present study was conducted to evaluate the insecticidal effect of different essential oils in combination with a detergent, and to identify their major bioactive components for the control of *D. opuntiae* nymphs and adult females. Among all the EOs tested, *M. pulegium* L and *O. vulgare* at 5% after two successive sprays, and their combination with black soap at 60 g/L, were the most effective against different stages of *D. opuntiae*. The insecticidal activity of essentials oils depends on plants derived, concentration of EOs (tested), and their chemical composition, as well as on the exposure time.

The results of GC-MS analysis of *M. pulegium* L. oil revealed the predominance of a monoterpene ketone, pulegone with (84.69%), which is known for its insecticidal effect against numerous insect pests [40,41]. Pulegone was found to be the major component of the Moroccan *M. pulegium* oil, with 73.33% and 73.44% [42,43]. Similarly, Boutabia et al. [44] reported pulegone as the major component of *M. pulegium* with (61.24%) in Algeria. Monoterpenoids such as pulegone destroy insects by a nervous system dysfunction or neuromuscular action [45,46,47]. According to Tong et al. [48], pulegone are positive allosteric modulators for the American cockroach and housefly GABA receptors. They bind to GABA receptors associated with Cl channels—located on the membrane of post-synaptic neurons—and therefore disrupt the function of GABA synapses, causing inhibitory effects on the nervous system of insects. Furthermore, pulegone has a neurotoxin effect on insects by inhibiting acetylcholinesterase activity [49].

The GC-MS analysis showed a wide range of chemical compounds in *O. vulgare* oil, with durenol (76.53%) as the most abundant compounds. A recent study reported the carvacrol as the most abundant compound of *O. vulgare* oil, with 57.3% [50]. Similarly, Xie et al. [51] also reported that carvacrol (58.13%) was the main compound of *O. vulgare* oil. The variations in chemical composition of different essential oils across countries have been attributed to environmental factors (nutrition/climate/weather/soil) that can influence the regulation of the essential oil biosynthesis, in addition to intrinsic factors (plant material and physiological and genetic differences) [52,53].

The results showed that the insecticidal activity of *M. pulegium* L. and *O. vulgare* L. under controlled conditions increased with increasing concentrations for different exposure times. However, the insecticidal effect of the tested oils appeared to be more effective when they were used in a double application or in combination with black soap (60 g/L) under field conditions, without any evident phytotoxicity for the treated plants. In this context, the efficacy of using two sprays of essential oil was confirmed by Boulamtat et al. [54], who showed a significant effect of double application of *O. basilicum* oil at 2.5% in controlling chickpea pod borer (*Helicoverpa armigera*) (Lepidoptera: Noctuidae) larvae under field conditions.

Several authors have reported that *M. pulegium* has a strong insecticidal effect on a range of insect pests. Franzios et al. [40] reported that the important insecticidal effect of *M. pulegium* is due mainly to its main constituent, pulegone. Miguel et al. [55] reported that *M. pulegium* induced 100% mortality to *Ceratitis capitata* (Diptera: Tephritidae) with 0.3% (*w/v*) EO in mucilage at 72 h after treatment. Similarly, *M. pulegium* oil exhibited greater toxicity (100%) against *Sitophilus oryzae* (Coleoptera: Curculionidae) and *Tribolium castaneum* (Coleoptera: Tenebrionidae) adults with 0.16 μL/cm^2^ at 24 h [56]_._ In addition, several studies have also reported the toxicity of *M. pulegium* against various insects such as Hessian fly *Mayetiola destructor* (Diptera: Cecidomyiidae) [57], *Bactrocera oleae* (Diptera: Tephritidae) [41], *Bemisia tabaci* (Hemiptera: Aleyrodidae) [58], *Camptomyia corticalis* (Diptera: Cecidomyiidae) [59], *Sitophilus zeamais* (Coleoptera: Curculionidae) [60], and *Helicoverpa armigera* (Lepidoptera: Noctuidae) [61].

Comparison of insecticidal efficacy of the different oils showed that the *O. vulgare* at 5% was the second most effective on *D. opuntiae* adult females and nymphs under controlled conditions and in the field. Several studies have reported the high insecticidal effects of *O. vulgare* on a range of insect pests, including storage pests (*Tribolium castaneum* and *Sitophilus oryzae* [62,63], the housefly (*Musca domestica*) (Diptera: Muscidae) [51,64], and the cotton leaf worm (*Spodoptera littoralis*) (Lepidoptera: Noctuidae) [65]. Govindarajan et al. [66] confirmed the excellent larvicidal potential of carvacrol and terpinen-4-ol as major constituents from the essential oil of *O. vulgare* on different mosquitoes (*Anopheles stephensi* (Diptera: Culicidae), *A. subpictus* (Diptera: Culicidae), *Culex quinquefasciatus* (Diptera: Culicidae), and *C. tritaeniorhynchus* (Diptera: Culicidae)). However, the current study showed durenol as the major bioactive component of *O. vulgare*, which could be responsible for the insecticidal properties against different stages of *D. opuntiae*. However, the exact mechanism of action of this molecule is still unknown. 

On the other hand, the application of the essential oils in combination with the detergent black soap at 60 g/L showed a high insecticidal effect against *D. opuntiae* nymphs and females. Black soap is a traditional product manufactured from natural fatty acids derived from olive oil. Soaps and oils may have been among the first treatments used to control insects [67]. *M. pulegium* and *O. vulgare* at 5% combined with black soap at 60 g/L showed the greatest mortality of females under field conditions with 100% at 3 days after second application. The first application of black soap is used to remove the thicker wax, which resulted in the exposure of females and nymphs of *D. opuntiae* to high contact toxicity of the tested oils *M. pulegium* and *O. vulgare*. The present study corroborates the findings of [9] using the black soap solution at 60 g/L in combination with *C. annuum* fruit extract at 200 g/L. This combination showed an excellent control of *D. opuntiae* females with 87.31% at 7 days after application. However, the application of the extract *C. annum* alone at 200 g/L showed less mortality of females with 18.40%. The same study reported that the double application of black soap at 60 g/L over a 3-day spray interval significantly increased the mortality of adult females up to 82.5% at 3 days after the second application [9]. The lethal mechanisms involved in use of soaps and detergents are the wax removal, repellency or cell membrane disruption, arthropod dislodging, and drowning [68,69].

## 5. Conclusions

The findings of the current study show that double applications of *M. pulegium* and *O. vulgare* oils alone at 5% or in combination with black soap at 60 g/L could be used as one of the IPM components for the control of *D. opuntiae* as an effective alternative to synthetic pesticides. Further studies are needed to develop formulations based on the bioactive molecules pulegone and durenol as new and more effective biopesticides against *D. opuntiae*.

## Figures and Tables

**Table 1:** Mean percentage mortality ±SE of *D*. *opuntiae* adult females after exposure to different essential oils.

Treatments	Concentrations (%)	Mortality (%)					
		3 d	4 d	5 d	6 d	7 d	8 d
*O. vulgare*	5	66.00 ± 2.45 ^f^	80.00 ± 0.00 ^f^	92.00 ± 2.00 ^g^	92.00 ± 2.00 ^gh^	92.00 ± 2.00 ^ij^	92.00 ± 2.00 ^hi^
	2.5	40.00 ± 3.16 ^e^	58.00 ± 2.00 ^e^	66.00 ± 2.45 ^ef^	80.00 ± 0.00 ^efg^	82.00 ± 2.00 ^ghi^	82.00 ± 2.00 ^gh^
	1.25	12.00 ± 2.00 ^c^	18.00 ± 2.00 ^c^	30.00 ± 4.47 ^d^	52.00 ± 4.90 ^c^	52.00 ± 4.90 ^ef^	52.00 ± 4.90 ^ef^
	0.625	0.00 ± 0,00 ^a^	0.00 ± 0.00 ^a^	0.00 ± 0.00 ^a^	4.00 ± 2.45 ^a^	4.00 ± 2.45 ^ab^	4.00 ± 2.45 ^ab^
*M. pulegium*	5	74.00 ± 2.45 ^f^	80.00 ± 0.00 ^f^	98.00 ± 2.00 ^g^	98.00 ± 2.00 ^h^	98.00 ± 2.00 ^j^	98.00 ± 2.00 ^i^
	2.5	30.00 ± 0.00 ^d^	58.00 ± 4.47 ^e^	68.00 ± 4.47 ^ef^	86.00 ± 5.48 ^fg^	86.00 ± 5.48 ^hi^	88.00 ± 4.47 ^h^
	1.25	0.00 ± 0.00 ^a^	0.00 ± 0.00 ^a^	18.00 ± 3.74 ^cd^	24.00 ± 2.45 ^b^	24.00 ± 2.45 ^cd^	26.00 ± 2.45 ^cd^
	0.625	0.00 ± 0.00 ^a^	0.00 ± 0.00 ^a^	0.00 ± 0.00 ^a^	0.00 ± 0.00 ^a^	0.00 ± 0.00 ^a^	0.00 ± 0.00 ^a^
*T. vulgaris*	5	4.00 ± 2.45 ^b^	42.00 ± 3.74 ^d^	50.00 ± 3.16 ^e^	66.00 ± 5.10 ^cde^	74.00 ± 2.45 ^fgh^	80.00 ± 3.16 ^gh^
	2.5	0.00 ± 0.00 ^a^	8.00 ± 2.00 ^b^	26.00 ± 2.45 ^cd^	56.00 ± 2.45 ^cd^	64.00 ± 2.45 ^fg^	64.00 ± 2.45 ^fg^
	1.25	0.00 ± 0.00 ^a^	0.00 ± 0.00 ^a^	6.00 ± 2.45 ^ab^	14.00 ± 2.45 ^b^	28.00 ± 2.00 ^d^	32.00 ± 2.00 ^de^
	0.625	0.00 ± 0.00 ^a^	0.00 ± 0.00 ^a^	2.00 ± 2.00 ^a^	4.00 ± 2.45 ^a^	12.00 ± 3.74 ^bc^	12.00 ± 3.74 ^bc^
*R. officinalis*	5	0.00 ± 0.00 ^a^	32.00 ± 2.00 ^d^	60.00 ± 0.00 ^ef^	76.00 ± 2.45 ^def^	76.00 ± 2.45 ^gh^	82.00 ± 2.00 ^gh^
	2.5	0.00 ± 0.00 ^a^	0.00 ± 0.00 ^a^	12.00 ± 2.00 ^bc^	16.00 ± 4.00 ^b^	38.00 ± 7.35 ^de^	38.00 ± 7.35 ^de^
	1.25	0.00 ± 0.00 ^a^	0.00 ± 0.00 ^a^	4.00 ± 2.45 ^a^	4.00 ± 2.45 ^a^	4.00 ± 2.45 ^ab^	4.00 ± 2.45 ^ab^
	0.625	0.00 ± 0.00 ^a^	0.00 ± 0.00 ^a^	0.00 ± 0.00 ^a^	0.00 ± 0.00 ^a^	0.00 ± 0.00 ^a^	0.00 ± 0.00 ^a^
*M. communis*	5	0.00 ± 0.00 ^a^	54.00 ± 2.45 ^e^	70.00 ± 0.00 ^f^	80.00 ± 0.00 ^efg^	80.00 ± 0.00 ^ghi^	80.00 ± 0.00 ^gh^
	2.5	0.00 ± 0.00 ^a^	20.00 ± 4.47 ^c^	20.00 ± 4.47 ^cd^	20.00 ± 4.47 ^b^	20.00 ± 4.47 ^cd^	20.00 ± 4.47 ^cd^
	1.25	0.00 ± 0.00 ^a^	0.00 ± 0.00 ^a^	0.00 ± 0.00 ^a^	0.00 ± 0.00 ^a^	0.00 ± 0.00 ^a^	0.00 ± 0.00 ^a^
	0.625	0.00 ± 0.00 ^a^	0.00 ± 0.00 ^a^	0.00 ± 0.00 ^a^	0.00 ± 0.00 ^a^	0.00 ± 0.00 ^a^	0.00 ± 0.00 ^a^
*E. globulus*	5	0.00 ± 0.00 ^a^	0.00 ± 0.00 ^a^	0.00 ± 0.00 ^a^	0.00 ± 0.00 ^a^	0.00 ± 0.00 ^a^	0.00 ± 0.00 ^a^
	2.5	0.00 ± 0.00 ^a^	0.00 ± 0.00 ^a^	0.00 ± 0.00 ^a^	0.00 ± 0.00 ^a^	0.00 ± 0.00 ^a^	0.00 ± 0.00 ^a^
	1.25	0.00 ± 0.00 ^a^	0.00 ± 0.00 ^a^	0.00 ± 0.00 ^a^	0.00 ± 0,00 ^a^	0.00 ± 0.00 ^a^	0.00 ± 0.00 ^a^
	0.625	0.00 ± 0.00 ^a^	0.00 ± 0.00 ^a^	0.00 ± 0.00 ^a^	0.00 ± 0.00 ^a^	0.00 ± 0.00 ^a^	0.00 ± 0.00 ^a^
Check (Water)		0.00 ± 0.00 ^a^	0.00 ± 0.00 ^a^	0.00 ± 0.00 ^a^	0.00 ± 0.00 ^a^	0.00 ± 0.00 ^a^	0.00 ± 0.00 ^a^
*p*-value		<0.001	<0.001	<.0.001	<0.001	<0.001	<0.001

Means in the same column followed by different letter(s) are significantly different based on Tukey test (*p* < 0.05).

**Table 2:** Mean percentage ±SE of *D. opuntiae* nymphs mortality after exposure to different essential oils.

Treatments	Concentrations (%)	Mortality (%)				
		3 h	6 h	24 h	48 h	72 h
*O. vulgare*	5	86.00 ± 2.45 ^j^	86.00 ± 2.45 ^i^	98.00 ± 2.00 ^l^	98.00 ± 2.00 ^i^	98.00 ± 2.00 ^lm^
	2.5	52.00 ± 2.00 ^gh^	66.00 ± 2.45 ^h^	76.00 ± 2.00 ^jk^	78.00 ± 2.00 ^fg^	78.00 ± 2.00 ^hij^
	1.25	12.00 ± 3.74 ^c^	12.00 ± 3.74 ^cd^	34.00 ± 5.10 ^efgh^	58.00 ± 2.00 ^ef^	58.00 ± 2.00 ^defg^
	0.625	0.00 ± 0.00 ^a^	0.00 ± 0.00 ^a^	10.00 ± 0.00 ^bcd^	30.00 ± 3.16 ^bcd^	48.00 ± 2.00 ^cdef^
*M. pulegium*	5	74.00 ± 4.00 ^ij^	98.00 ± 2.00 ^j^	98.00 ± 2.00 ^l^	98.00 ± 2.00 ^i^	98.00 ± 2.00 ^lm^
	2.5	36.00 ± 4.00 ^efg^	42.00 ± 4.90 ^fg^	68.00 ± 3.74 ^ij^	92.00 ± 2.00 ^ghi^	92.00 ± 2.00 ^kl^
	1.25	16.00 ± 2.45 ^cd^	18.00 ± 2.00 ^de^	38.00 ± 2.00 ^fgh^	66.00 ± 2.45 ^ef^	72.00 ± 2.00 ^ghi^
	0.625	0.00 ± 0.00 ^a^	2.00 ± 2.00 ^ab^	16.00 ± 4.00 ^bcde^	26.00 ± 2.45 ^bc^	32.00 ± 2.00 ^abc^
*T. vulgaris*	5	78.00 ± 2.00 ^ij^	78.00 ± 2.00 ^hi^	88.00 ± 2.00 ^k^	96.00 ± 2.45 ^hi^	100.00 ± 0.00 ^m^
	2.5	62.00 ± 2.00 ^hi^	62.00 ± 2.00 ^gh^	78.00 ± 2.00 ^jk^	88.00 ± 2.00 ^gh^	88.00 ± 2.00 ^ijk^
	1.25	8.00 ± 2.00 ^bc^	20.00 ± 0.00 ^def^	50.00 ± 3.16 ^hi^	64.00 ± 2.45 ^ef^	68.00 ± 2.00 ^fgh^
	0.625	2.00 ± 2.00 ^ab^	10.00 ± 3.16 ^bcd^	26.00 ± 2.45 ^defg^	32.00 ± 3.74 ^bcd^	44.00 ± 2.45 ^bcd^
*R. officinalis*	5	44.00 ± 4.00 ^fgh^	56.00 ± 4.00 ^gh^	64.00 ± 4.00 ^ij^	88.00 ± 3.74 ^ghi^	88.00 ± 3.74 ^jk^
	2.5	24.00 ± 2.45 ^de^	32.00 ± 2.00 ^ef^	44.00 ± 2.45 ^ghi^	58.00 ± 2.00 ^ef^	66.00 ± 2.45 ^efgh^
	1.25	18.00 ± 2.00 ^cd^	18.00 ± 2.00 ^de^	20.00 ± 3.16 ^cdef^	26.00 ± 4.00 ^bc^	46.00 ± 4.00 ^bcde^
	0.625	0.00 ± 0.00 ^a^	0.00 ± 0.00 ^a^	8.00 ± 2.00 ^abc^	16.00 ± 2.45 ^ab^	30.00 ± 4.47 ^abc^
*M. communis*	5	28.00 ± 2.00 ^def^	56.00 ± 2.45 ^gh^	68.00 ± 3.74 ^ij^	90.00 ± 3.16 ^ghi^	90.00 ± 3.16 ^jk^
	2.5	8.00 ± 2.00 ^bc^	18.00 ± 2.00 ^de^	30.00 ± 0.00 ^efgh^	54.00 ± 2.45 ^def^	72.00 ± 2.00 ^ghi^
	1.25	2.00 ± 2.00 ^ab^	6.00 ± 2.45 ^abc^	20.00 ± 4.47 ^cdef^	46.00 ± 2.45 ^cde^	50.00 ± 0.00 ^cdef^
	0.625	0.00 ± 0.00 ^a^	0.00 ± 0.00 ^a^	6.00 ± 2.45 ^ab^	16.00 ± 2.45 ^ab^	16.00 ± 2.45 ^a^
*E. globulus*	5	0.00 ± 0.00 ^a^	18.00 ± 2.00 ^de^	34.00 ± 5.10 ^efgh^	58.00 ± 2.00 ^ef^	66.00 ± 2.45 ^efgh^
	2.5	0.00 ± 0.00 ^a^	6.00 ± 2.45 ^abc^	20.00 ± 4.47 ^cdef^	44.00 ± 4.00 ^cde^	50.00 ± 3.16 ^cdef^
	1.25	0.00 ± 0.00 ^a^	0.00 ± 0.00 ^a^	14.00 ± 2.45 ^bcde^	22.00 ± 2.00 ^bc^	40.00 ± 3.16 ^bcd^
	0.625	0.00 ± 0.00 ^a^	0.00 ± 0.00 ^a^	2.00 ± 2.00 ^a^	8.00 ± 3.74 ^a^	26.00 ± 2.45 ^ab^
Check (Water)		0.00 ± 0.00 ^a^	0.00 ± 0.00 ^a^	0.00 ± 0.00. ^a^	0.00 ± 0.00 ^a^	0.00 ± 0.00 ^a^
*p*-value		<0.001	<0.001	<0.001	<0.001	<0.001

Means in the same column followed by different letter(s) are significantly different based on Tukey test (*p* < 0.05).

**Table 3:** Chemical composition of *M. pulegium* L., *O. vulgare* L., *R. officinalis* L., and *M. communis* obtained by GC and identified by mass spectroscopy.

No	Compounds	Compounds Percentage (%)			
		*M. pulegium* L.	*R. officinalis* L.	*O. vulgare* L.	*M. communis*
1	alpha.-pinene	-	-	-	0.14
2	alpha.-terpineol	-	4.44	-	-
3	alpha.-terpinyl acetate	-	-	-	0.24
4	beta.-bisabolene	-	-	-	0.27
5	beta.-myrcene	-	-	-	0.29
6	beta.-ocimene	-	-	-	0.17
7	beta.-pinene	-	-	-	0.5
8	(-)-β-pinene	-	-	-	16.94
9	myrtenol	-	-	-	0.68
10	2-bornanone	-	24.74	-	-
11	bornyl acetate	-	2.09	-	4.96
12	butyric acid, 2-methyl-	-	-	-	0.38
13	geraniol butyrate	-	-	-	0.35
14	camphene	-	-	-	0.09
15	caryophyllene	-	8.98	1.93	0.4
16	caryophyllene oxide	-	-	-	0.11
17	terpinolene	-	-	-	0.26
18	d-limonene	1.69	-	-	-
19	6,6-imethylbicyclo[3.1.1]hept-2-en-2-yl)methyl ethyl carbonate	-	-	-	0.37
20	durenol	-	-	76.53	-
21	durohydroquinone	-	-	-	0.35
22	endo-borneol	-	7.53	-	-
23	estragole	-	-	-	0.26
24	eucalyptol	-	45.16	-	42.6
25	gamma.-terpinene	-	1.37	10.25	0.22
26	geranyl acetate	-	-	-	3.18
27	humulene	2.02	1.01	-	0.26
28	linalool	-	-	-	3.68
29	linalyl acetate	-	-	-	0.1
30	menthene	-	0.69	-	-
31	methyleugenol	-	-	-	1.22
32	1-methyl-4-ethylaminocytosine	1.25	-	-	-
33	3-menthene	0.52	-	-	-
34	myrtenyl acetate	-	-	-	18.23
35	cis-Geraniol	-	-	-	0.27
36	hydroxycineyl acetate	-	-	-	0.56
37	p-cymene	-	-	7.75	-
38	piperitenone	2.05	-	-	-
39	isobutyl isobutyrate	-	-	-	0.29
40	pulegone	84.69	-	-	-
41	terpinen-4-ol	-	1.31	-	0.49
42	trans-pinocarvyl acetate	-	-	-	0.41
43	Compounds not identified	7.78	2.68	3.54	1.73
	Total	92.22	97.32	96.46	98.27

**Table 4:** Insecticidal effects of EOs and their combination with black soap on *D. opuntiae* nymphs.

Treatments/Exposure Period	% Mortality of Nymphs after First Spray				% Mortality of Nymphs after Second Spray			
	1 d	3 d	5 d	7 d	1 d	3 d	5 d	7 d
*M. pulegium* (5%) + black soap (60 g/L)	90.00 ± 0.00 ^e^	96.67 ± 1.60 ^e^	100.00 ± 0.00 ^e^	100.00 ± 0.00 ^e^	100.00 ± 0.00 ^d^	100.00 ± 0.00 ^c^	100.00 ± 0.00 ^b^	100.00 ± 0.00 ^b^
*O. vulgare* (5%) + black soap (60 g/L)	81.11 ± 1.11 ^d^	87.78 ± 1.40 ^d^	92.22 ± 1.40 ^d^	92.22 ± 1.40 ^d^	100.00 ± 0.00 ^d^	100.00 ± 0.00 ^c^	100.00 ± 0.00 ^b^	100.00 ± 0.00 ^b^
*R. officinalis*(5%) + black soap (60 g/L)	70.00 ± 0.00 ^c^	78.89 ± 1.11 ^bcd^	84.44 ± 1.76 ^bcd^	84.44 ± 1.76 ^bcd^	94.44 ± 1.70 ^c^	100.00 ± 0.00 ^c^	100.00 ± 0.00 ^b^	100.00 ± 0.00 ^b^
*M. communis* (5%) + black soap(60 g/L)	65.56 ± 1.76 ^c^	70.08 ± 1.67 ^b^	71.19 ± 1.47 ^b^	73.33 ± 1.11 ^b^	88.89 ± 1.11 ^b^	97.78 ± 1.47 ^c^	98.89 ± 1.11 ^b^	98.89 ± 1.11 ^b^
*M. pulegium* (5%)	87.78 ± 1.47 ^e^	93.33 ± 1.67 ^e^	98.89 ± 1.11 ^e^	98.89 ± 1.11 ^e^	100.00 ± 0.00 ^d^	100.00 ± 0.00 ^c^	100.00 ± 0.00 ^b^	100.00 ± 0.00 ^b^
*O. vulgare* (5%)	77.78 ± 1.47 ^d^	85.56 ± 1.76 ^cd^	87.78 ± 1.47 ^cd^	90.00 ± 0.00 ^cd^	100.00 ± 0.00 ^d^	100.00 ± 0.00 ^c^	100.00 ± 0.00. ^b^	100.00 ± 0.00 ^b^
*R. officinalis* (5%)	70.00 ± 0.00 ^c^	75.56 ± 1.76 ^bc^	78.89 ± 1.11 ^bc^	80.00 ± 0.00 ^bc^	90.00 ± 0.00 ^b^	98.89 ± 1.11 ^c^	98.89 ± 1.11 ^b^	98.89 ± 1.11 ^b^
*M. communis* (5%)	60.00 ± 0.00 ^b^	67.78 ± 1.47 ^b^	73.33 ± 1.67 ^bc^	74.44 ± 1.76 ^b^	83.33 ± 1.67 ^b^	93.33 ± 1.67 ^b^	96.67 ± 1.67 ^b^	96.67 ± 1.67 ^b^
Check	0.00 ± 0.00 ^a^	0.00 ± 0.00 ^a^	0.00 ± 0.00 ^a^	0.00 ± 0.00 ^a^	0.00 ± 0.00 ^a^	0.00 ± 0.00 ^a^	0.00 ± 0.00 ^a^	0.00 ± 0.00 ^a^
*p* value	<0.001	<0.001	<0.001	<0.001	<0.001	<0.001	<0.001	<0.001

Means in the same column followed by different letter(s) are significantly different based on Tukey test (*p* < 0.05).

**Table 5:** Insecticidal effects of EOs and their combination with black soap on *D. opuntiae* females.

Treatments/Exposure Period	% Mortality of Females after First Spray				% Mortality of Females after Second Spray			
	1 d	3 d	5 d	7 d	1 d	3 d	5 d	7 d
*M. pulegium* (5%) + black soap (60 g/L)	76.33 ± 0.67 ^g^	87.11 ± 0.51 ^h^	93.78 ± 0.91 ^h^	96.33 ± 0.41 ^h^	99.89 ± 0.11 ^g^	100.00 ± 0 ^f^	100.00 ± 0.00 ^f^	100.00 ± 0.00 ^f^
*O. vulgare* (5%) + black	73.30 ± 0.96 ^f^	82.11 ± 0.75 ^g^	90.44 ± 0.41 ^g^	92.56 ± 0.58 ^g^	99.56 ± 0.24 ^g^	100.00 ± 0 ^f^	100.00 ± 0 ^f^	100.00 ± 0.00 ^f^
*R. officinalis* (5%) + black soap (60 g/L)	56.00 ± 0.62 ^e^	61.33 ± 0.55 ^f^	66.78 ± 0.78 ^f^	69.89 ± 0.56 ^f^	79.44 ± 0.41 ^f^	84.56 ± 0.73 ^e^	85.00 ± 0.71 ^d^	85.00 ± 0.71 ^d^
*M. communis* (5%) + black soap (60 g/L)	51.44 ± 0.71 ^d^	58.56 ± 0.67 ^e^	63.00 ± 0.58 ^e^	65.78 ± 0.62 ^e^	73.11 ± 0.93 ^e^	78.67 ± 0.44 ^d^	79.56 ± 0.78 ^c^	79.56 ± 0.78 ^c^
*M. pulegium* (5%)	25.11 ± 0.42 ^c^	37.78 ± 0.94 ^d^	51.00 ± 0.53 ^d^	54.11 ± 0.45 ^d^	76.44 ± 0.60 ^ef^	84.78 ± 0.43 ^e^	91.00 ± 1.14 ^e^	91.00 ± 1.14 ^e^
*O. vulgare* (5%)	22.89 ± 0.90 ^c^	26.78 ± 0.72 ^c^	31.22 ± 0.55 ^c^	33.56 ± 0.58 ^c^	61.67 ± 1.08 ^d^	77.44 ± 0.78 ^d^	81.89 ± 0.51 ^cd^	81.89 ± 0.51 ^cd^
*R. officinalis* (5%)	15.44 ± 0.69 ^b^	19.22 ± 0.76 ^b^	22.33 ± 0.71 ^b^	25.78 ± 0.64 ^b^	45.33 ± 1.30 ^c^	62.11 ± 0.98 ^c^	64.78 ± 0.89 ^b^	64.78 ± 0.89 ^b^
*M. communis* (5%)	15.11 ± 0.59 ^b^	18.89 ± 0.65 ^b^	22.11 ± 0.61 ^b^	25.56 ± 0.53 ^b^	36.78 ± 0.83 ^b^	58.89 ± 0.56 ^b^	62.56 ± 0.63 ^b^	62.56 ± 0.63 ^b^
Check	0.00 ± 0 ^a^	0.00 ± 0.00 ^a^	0.00 ± 0.00 ^a^	0.00 ± 0.00 ^a^	0.00 ± 0.00 ^a^	0.00 ± 0.00 ^a^	0.00 ± 0.00 ^a^	0.00 ± 0.00 ^a^
*p* value	<0.001	<0.001	<0.001	<0.001	<0.001	<0.001	<0.001	<0.001

Means in the same column followed by different letter(s) are significantly different based on Tukey test (*p* < 0.05).

## Data Availability

The following are available online at https://doi.org/10.5281/zenodo.5606066 (accessed on 18 September 2021). Figure S1, Chromatogram obtained by GC-MS of the four essential oils (*Mentha pulegium*, *Origanum vulgare*, *Rosmarinus officinalis*, *Myrtus communis*).
